# The Metabolomic Signature of Opa1 Deficiency in Rat Primary Cortical Neurons Shows Aspartate/Glutamate Depletion and Phospholipids Remodeling

**DOI:** 10.1038/s41598-019-42554-7

**Published:** 2019-04-15

**Authors:** Juan Manuel Chao de la Barca, Macarena S. Arrázola, Cinzia Bocca, Laetitia Arnauné-Pelloquin, Olga Iuliano, Guillaume Tcherkez, Guy Lenaers, Gilles Simard, Pascale Belenguer, Pascal Reynier

**Affiliations:** 1 Equipe Mitolab, Institut MITOVASC, CNRS 6015, INSERM U1083, Université d’Angers, Angers, France; 2 Département de Biochimie et Génétique, Centre Hospitalier Universitaire, Angers, France; 3 Centre de Recherches sur la Cognition animale (CRCA), Centre de Biologie Intégrative (CBI), Université de Toulouse, CNRS, UPS, Toulouse, France; 4 Present Address: Center for Integrative Biology, Facultad de Ciencias, Universidad Mayor, Santiago, Chile; 5 Research School of Biology, The Australian National University, Canberra, 2601 ACT Australia

## Abstract

Pathogenic variants of *OPA1*, which encodes a dynamin GTPase involved in mitochondrial fusion, are responsible for a spectrum of neurological disorders sharing optic nerve atrophy and visual impairment. To gain insight on OPA1 neuronal specificity, we performed targeted metabolomics on rat cortical neurons with OPA1 expression inhibited by RNA interference. Of the 103 metabolites accurately measured, univariate analysis including the Benjamini-Hochberg correction revealed 6 significantly different metabolites in OPA1 down-regulated neurons, with aspartate being the most significant (*p* < 0.001). Supervised multivariate analysis by OPLS-DA yielded a model with good predictive capability (Q^2^_cum_ = 0.65) and a low risk of over-fitting (permQ2 = −0.16, CV-ANOVA *p*-value 0.036). Amongst the 46 metabolites contributing the most to the metabolic signature were aspartate, glutamate and threonine, which all decreased in OPA1 down-regulated neurons, and lysine, 4 sphingomyelins, 4 lysophosphatidylcholines and 32 phosphatidylcholines which were increased. The phospholipid signature may reflect intracellular membrane remodeling due to loss of mitochondrial fusion and/or lipid droplet accumulation. Aspartate and glutamate deficiency, also found in the plasma of OPA1 patients, is likely the consequence of respiratory chain deficiency, whereas the glutamate decrease could contribute to the synaptic dysfunction that we previously identified in this model.

## Introduction

OPA1 is a dynamin GTPase controlling the mitochondrial network and cristae organization through its role in the inner mitochondrial membrane structuration^1^. More than 300 *OPA1* gene variants have been reported since its first description as the main contributor to dominant optic atrophies (DOA, MIM#165500)^2–4^. Clinical studies have progressively shown that OPA1 dysfunction was responsible for a large spectrum of neurological disorders involving optic, auditory and peripheral nerves as well as the brain^5,6^. This has highlighted the importance of OPA1, and more generally of mitochondrial dynamics, in neuronal plasticity^7^.

OPA1 inactivation affects many functions, such as mitochondrial fusion, mitochondrial cristae organization and cell death^8,9^, oxidative phosphorylation (OXPHOS) and energy production^10^, mitochondrial DNA maintenance^11–13^, calcium fluxes^14^, reactive oxygen species production^15,16^, inflammation^17^, ageing^17,18^, mitophagy and mitochondrial renewal^18–20^.

To explore further the neuronal specificity characterizing OPA1 disorders, we have developed a rat primary neuron culture model, with a siRNA-induced OPA1 haploinsufficiency^21^, mimicking the common pathological mechanism of DOA. This model allowed us disclosing dendritic and synaptic impairments associated with altered mitochondrial respiration and reduced abundance of mitochondria along dendrites^16,21^. Accordingly, synaptic defects and dendropathy were also found *in vivo* in retinal ganglion cells (RGC) from a DOA mouse model^22,23^. Furthermore, OPA1 loss induces pro-oxidative cellular conditions, leading to increased cell death^16^ and reduced mitophagy^24^ in rat cortical neurons.

Metabolomics is particularly useful to explore neuro-metabolic disorders. We recently used a non-targeted metabolomics approach on blood samples from OPA1 patients and showed a specific metabolic signature that includes the alteration of purine metabolism as well as that of amino acids and fatty acids^25^. We also applied targeted metabolomics on 9 tissues of 3 month old *Opa1*^*delTTAG/*+^ mice^18^, revealing a pre-symptomatic discriminant metabolomics signature in optic nerves, while other tissues were not affected at this age, thereby demonstrating the high sensitivity of optic nerve metabolism to OPA1 dysfunction^26^. In the later study, the metabolomics signature was characterized by a decrease in sphingomyelins and lysophosphatidylcholines, and an alteration of metabolites involved in neuro-protection and neuronal metabolism, suggesting myelin sheath alterations and axonal dysfunction, respectively.

In order to explore the neuronal specificity of the OPA1 clinical expression, we use here targeted metabolomics to characterize the metabolism of cultured rat cortical neurons in which OPA1 expression was inhibited by siRNA interference. In contrast to skin fibroblasts isolated from patients with similar OPA1 haploinsufficiency, the cortical neurons were considerably affected.

## Material and Methods

### Cell culture of rat embryonic cortical neurons

All animal procedures were approved by the “Animal Experimentation Ethics Committee” of the *Centre National de la Recherche Scientifique/Fédération de Recherche de Biologie de Toulouse* (C2EA-01) under the protocol number 01024–01.7. All experiments were performed in accordance with the relevant guidelines and regulations. Pregnant Wistar rats were delivered 48 h before sacrifice and kept with food and water *ad libitum*. Embryos were removed at Day 17 (D17) from pentobarbital anaesthetized females (Ceva Santé Animale, Libourne, France) and cortices were dissected as described^21^. Cortical cells (5 10^6^) obtained from D17 embryonic cortices were electroporated using the Rat Neuron Nucleofector® Kit (Amaxa®, Lonza, Basel, Switzerland) with either control luciferase-targeting (D-001210–02, Dharmacon, Lafayette, Colorado, USA) or OPA1-targeting (D-099769-01, Dharmacon) small interfering RNA (3 µg), leading to siLUC (controls) and siOPA1 cells. Transfected cells were plated on poly-D-lysine coated Petri dishes (1.25 10^6^ cells/35 mm) grown in Neurobasal A-25 medium supplemented with B27, L-glutamine, penicillin/streptomycin, amphotericin and lactic acid as described^21^. Cell pellets (3 10^6^) obtained after 9 days of culture were conserved at −80 °C until Western-blot and metabolomics analyses were performed.

### Western-Immuno-blotting

Proteins were extracted from primary cultured cortical neurons as described^16^. Protein extracts (10 µg) were subjected to SDS-PAGE and blotted onto nitrocellulose membranes, which were treated first with anti-OPA1 (1/500, BD Biosciences, Le Pont de Claix, France) and anti-GAPDH (1/500, Merck Millipore MAB374, Molsheim, France) primary antibodies, and then with horseradish peroxidase-conjugated secondary antibodies (1/5000, Abcam ab6789 and ab6721, Paris, France) as described^21^. After enhanced chemiluminescent detection, signals were analyzed using ChemiDoc^TM^ MP Imaging System and quantified by Image LabTM Software (Biorad®, Marnes la Coquette, France).

### Metabolite extraction

Each cell pellet (3 10^6^ cells, n = 10 for each siOPA1 and siLUC pairs) was dissolved in 100 µl of cold methanol/PBS (85:15, v/v). The mixture was transferred to a 0.5 ml homogenizer tube prefilled with ceramic beads. Cell lysis was achieved in a Precellys^®^24 homogenizer (Bertin instruments, Montigny-Le-Bretonneux, France) by two cycles of grinding (2 × 20 s at 6,500 rpm with a break of 20 s, followed by 30 s at 6,000 rpm) at 4 °C. The homogenate was centrifuged at 10,000 g for 5 min at 4 °C, the supernatant was then evaporated, and the resulting pellet stored at −80 °C until further analysis.

### Metabolomics analysis

Targeted quantitative metabolomics analyses were carried out as described^27^, using the Biocrates® Absolute IDQ p180 kit (Biocrates Life sciences AG, Innsbruck, Austria). This kit uses mass spectrometry (QTRAP 5500, SCIEX, Villebon-sur-Yvette, France) to quantify up to 188 different endogenous molecules (Supplementary Table S1). Flow injection analysis coupled with tandem mass spectrometry (FIA-MS/MS) was used to analyze carnitine, acylcarnitines, lipids and hexoses, whereas liquid chromatography (LC) was used to separate amino acids and biogenic amines before mass spectrometry quantitation.

All reagents used in this analysis were of LC-MS grade and purchased from VWR (Fontenay-sous-Bois, France) and Merck (Molsheim, France). Sample preparation and analysis were performed following the Kit User Manual. Briefly, after thawing on ice, the pellet was dissolved in 40 µL methanol. After vigorous mixing, 10 µL of each sample were added to a well of a 96-well plate. Metabolites were extracted and derivatized for the quantitation of amino acids and biogenic amines. Extracts were finally diluted with MS running solvent before FIA and LC-MS/MS analysis. Three quality controls (QCs) composed of three concentrations of a human plasma samples: low (QC1), medium (QC2) and high (QC3), were used to evaluate the performance of the analytical assay. A seven-point serial dilution of calibrators was added to the plate of the kit to generate calibration curves for the quantification of amino acids and biogenic amines.

### Statistical analyses

Raw data were examined before statistical analyses to exclude metabolites having more than 20% of their concentration values below the lower limit of quantitation (LLOQ) or above the upper limit of quantitation (ULOQ). To account for differences in metabolite concentration amongst samples merely due to differences in the number of cells used, each metabolite concentration belonging to a particular sample was divided by the sum of all the metabolites in that sample (row normalization).

Univariate analysis was performed using non-parametric Mann-Whitney-Wilcoxon test (thereafter simply referred to as ‘Wilcoxon test’) for quantitative variables and the Fischer exact test for qualitative variables. The Benjamini-Hochberg correction was used to compare multiple metabolite concentrations to keep the false discovery rate below 5%. Otherwise differences were considered significant when *p* ≤ 0.05.

Multivariate analysis was performed using SIMCA-P v.14.1 (Umetrics, Umeå, Sweden). Unsupervised principal component analysis (PCA) was employed to detect strong outliers and visualize sample clusters. A supervised method called orthogonal projection to latent structures by means of partial least square-discriminant analysis (OPLS-DA) was used to find the linear combination of metabolite concentration in the X (matrix of metabolites) space that best correlates to the Y response vector (binary classifier: siOPA1/siLUC). The quality of models was appreciated using two parameters: R² (goodness of fit) and Q²_cum_ (goodness of prediction). In the model with the best predictive capabilities (i.e., Q²_cum_ > 0.5), the risk of overfitting was measured by the predictive capabilities (Q²_cum-perm_) of the model obtained when elements of the response vector Y were randomly permuted (permutation test) (forming a new Y vector, Y_perm_). A non-over-fitted model is characterized by poor predictive capabilities (i.e., negative Q²_cum-perm_) when the permutation test yields models with no correlation between Y and Y_perm_. In the model retained, variables (here, metabolites) were selected according to their variable importance for the projection (VIP) and their loading rescaled as a correlation coefficient (p_cor_) between the original variable in the X matrix and the predictive component of interest. For a given metabolite *m* and a unique predictive component tp, VIP_*m*_ summarizes its importance for the OPLS-DA model, whereas p_cor,*m*_ indicates the magnitude of the correlation between *m* and tp. Thus, important variables (i.e., VIP > 1) having a high absolute p_cor_ value were retained since they were considered to be critical for group discrimination in predictive models. Plotting the VIP value *versus* p_cor_ for all variables (the “volcano” plot) enables visualization of the importance of each variable for group discrimination in the OPLS model.

In addition to the information obtained from individual metabolites, 25 sums and ratios of metabolites with biological significance have been calculated according to the RatioExplorer module provided by Biocrates® (Supplementary Table S2).

## Results

### OPA1 inactivation

**Figure 1:**
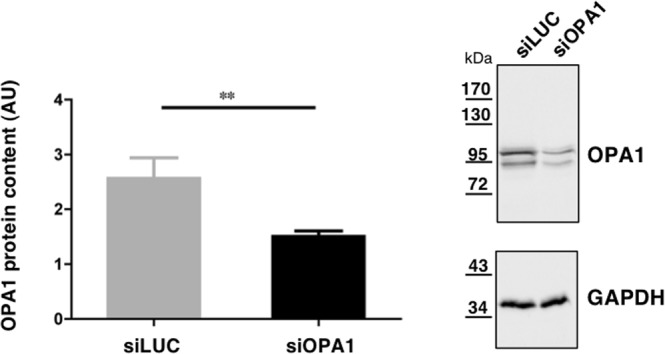
Box plot showing the OPA1 protein content relative to GAPDH protein in neurons transfected with siLUC or siOPA1, and cultured 9 days *in vitro*. Data represent the mean ± SEM of 10 independent experiments, and were statistically treated with Mann–Whitney **p < 0.01. AU = Arbitrary Units. One representative immunoblot is shown on the right panel.

To gain information on the impact of OPA1 inactivation on the metabolome of neuronal cells, we used embryonic rat cortical neurons grown in primary culture transfected with small interfering RNA against OPA1 (siOPA1; n = 10) or control small interfering RNA (siLUC; n = 10). After 9 days of culture *in vitro*, a Western-blot analysis using anti-OPA1 antibodies revealed that OPA1 expression, normalized to GAPDH, was reduced by 41.3% on average in siOPA1 compared with siLUC (p < 0.0029) (Fig. 1 and Supplementary Fig. S3).

### Univariate analysis

After validation of the kit plate based on QC samples, 103 (54.8%) metabolites were found to be accurately measured and consequently used for statistical analyses (Supplementary Table S4).

**Figure 2:**
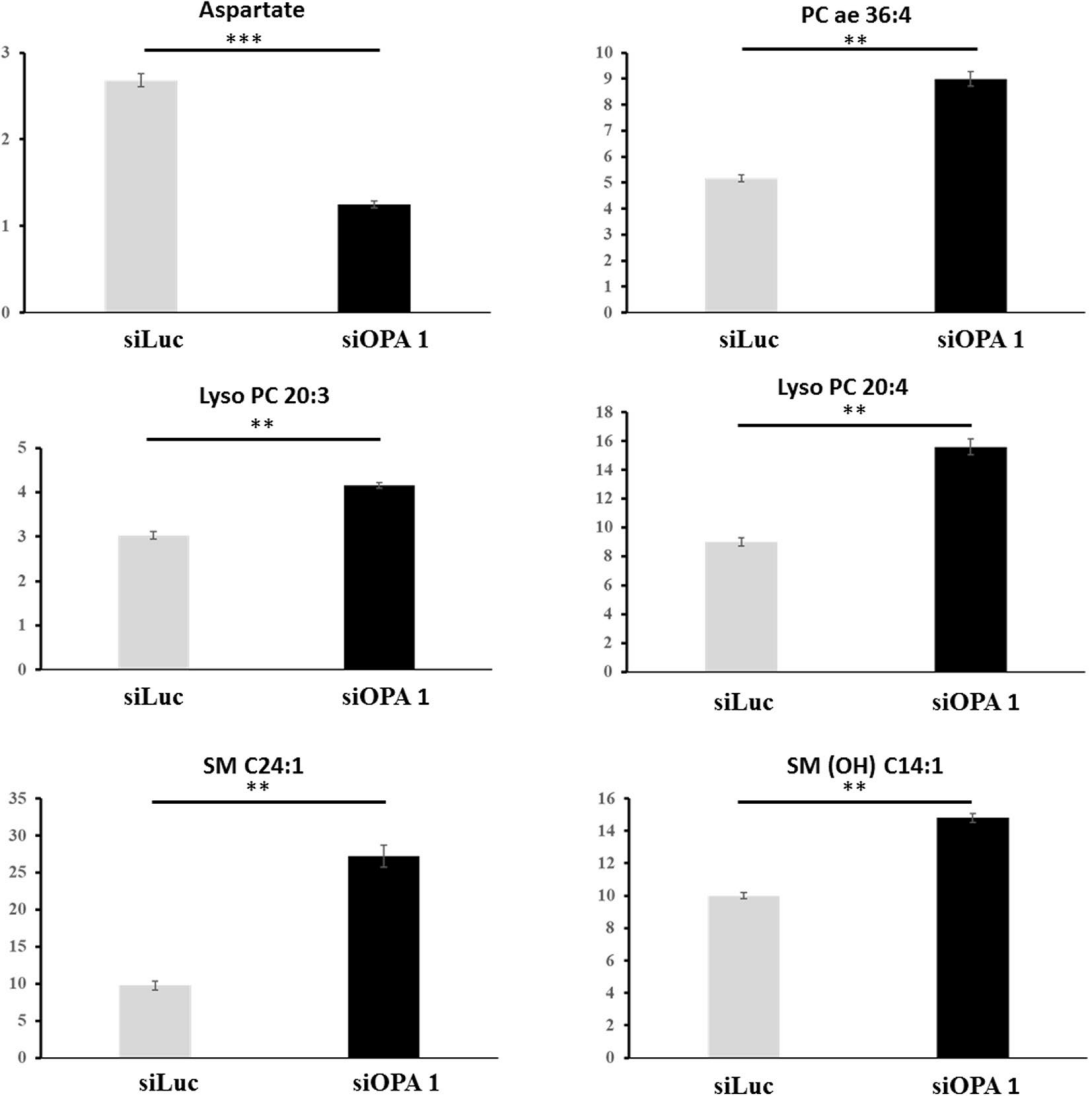
Box plot showing the 6 metabolites significantly different in siOPA1 (n = 10) compared to siLUC controls (n = 10) neurons using the univariate Wilcoxon test after Benjamini-Hochberg correction. Values in the y-axis have no dimension as they represent relative concentrations. ***p < 0.001; **p < 0.01. PC ae: Alkyl-acyl phosphatidylcholine; lysoPC: lysophosphatidylcholine; SM: sphingomyelin; SM(OH): hydroxy-sphingomyelin.

Univariate analysis using the Wilcoxon test showed that 36 metabolites were significantly different (*p* < 0.05) in siOPA1 compared to siLUC control neurons, while only 6 of them (decrease of aspartate and increase of lysoPC 20:3, lysoPC 20:4, SM 24:1, SM (OH) 14:1 and PC ae 36:4) remained significantly different after having applied the Benjamini-Hochberg correction with an overall false discovery rate (FDR) of 0.046 (Fig. 2).

### Multivariate analysis

**Figure 3:**
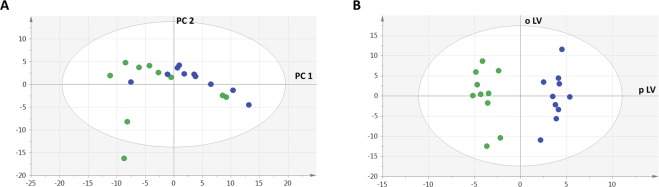
PCA (**A**) and OPLS-DA (**B**) scatter plots obtained from the matrix of metabolites for the 10 samples from siLUC (blue circles) and the 10 samples from siOPA1 (green circles). (**A**) PCA shows neither clear grouping nor outlier in the first principal plan, the green point appearing outside the ellipse being not a strong outlier according to Hotelling’s T2 range. (**B**) There is a clear between-group discrimination in the OPLS-DA plot along the predictive latent variable (p LV). Legend: PC1,2: Principal Components 1 and 2; o LV: first orthogonal latent variable; p LV: predictive latent variable.

Multivariate analysis by unsupervised principal component analysis (PCA) showed neither spontaneous grouping nor any strong outliers in the first principal plot (PC1 vs. PC2) (Fig. 3A). By contrast, supervised multivariate analysis by OPLS-DA showed a clear differentiation between siOPA1 and siLUC neurons, with high predictive capabilities (Q^2^_cumulated_ = 0.65) and good performances in the permutation test (R^2^X = 0.86 and R2Y = 0.95; Q2: (−0.16)), and the CV-ANOVA tests (*p-*value: 0.036) (Fig. 3B).

**Figure 4:**
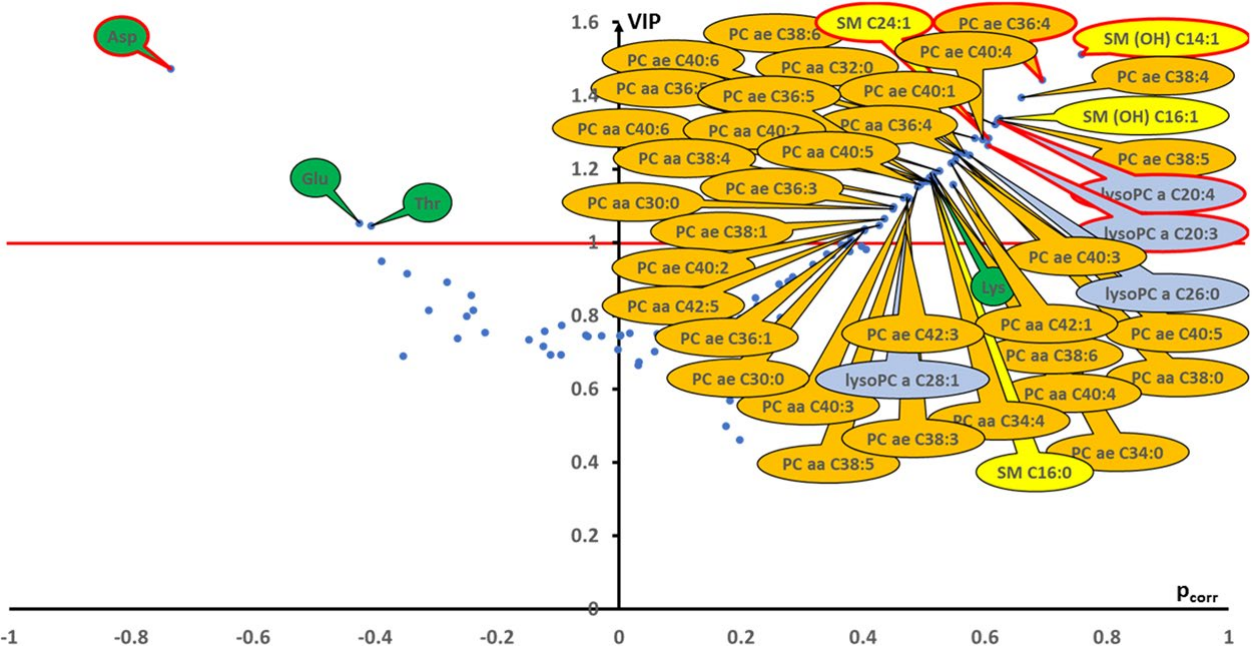
Volcano plot (p_corr_ vs. VIP) from the OPLS-DA model. Only the most discriminating metabolites having high VIP values ≥ 1 (indicated by the horizontal red line) have been labelled. Negative p_corr_ values (left) indicate diminished metabolite concentrations in siOPA1 neurons versus siLuc neurons, whereas positive p_corr_ values (right) indicate increased metabolite concentrations in siOPA1 neurons compared to the control group. The metabolomic signature accompanying the loss of OPA1 expression is associated to decreased levels of the amino acids (green bubbles) aspartate (Asp), glutamate (Glu) and threonine (Thr) and to increased levels of 34 phosphatidylcholines (PC, orange bubbles), 4 lysophosphatidylcholines (lysoPC, blue bubbles), 4 sphingomyelins (SM and SM(OH)), yellow bubbles) and the amino acid lysine (Lys, green bubble). Red-rimmed bubbles indicate metabolites significantly different between siOPA1 and siLUC neurons in univariate analysis (Wilcoxon test) after Benjamini-Hochberg correction.

The representation of VIP (variable importance for the projection) values against correlation coefficient (p_corr_) (volcano plot, Fig. 4) showed 46 important metabolites with a VIP value ≥ 1 and as such, considered as being crucial for group discrimination. Amongst these, aspartate, glutamate and threonine were decreased in siOPA1 compared to siLUC neurons, whereas 43 metabolites were increased. Lysine was the only increased amino acid while 4 sphingomyelins, 4 lysophosphatidylcholines and 34 phosphatidylcholines were all increased in siOPA1 neurons. Importantly, the 6 significant metabolites identified by the univariate analysis were the most important contributors of the 46 discriminating metabolites identified in the multivariate model.

Fourteen metabolites sums and/or ratios were calculated with available metabolite contents. Only unsaturated to saturated diacyl- and acyl-alkyl phosphatidylcholine (PC aa and PC ae, respectively) were significantly different between siOPA1 and siLUC neurons after Benjamini-Hochberg correction, with lower values for both ratios in siOPA1 neurons (Table 1).

**Table 1:** Metabolite sums and ratios calculated with amounts accurately determined and listed according to their P-value (from the smallest to the largest).

Ratio or sum	Fold Change (SiOPA1/ SiLUC)	Observed p-value	Corrected alpha threshold
UFA/SFA aa	0.68	**0.00021**	**0.0089**
UFA/SFA ae	0.83	**0.00209**	**0.0132**
SFA ae	1.66	0,02323	0.0170
SFA aa	1.66	0.02881	0.0204
Putrescine/Spermidine	1.23	0.05381	0.0236
Spermine/Spermidine	1.51	0.15641	0.0266
Methionine-SO/Methionine	1,14	0.19032	0.0294
Citrulline/Arginine	0.27	0.19280	0.0321
MUFA ae	1.27	0.24745	0.0348
PUFA ae	1.20	0.24745	0.0374
Total lysoPC/ Total PC	0.75	0.24745	0.0399
MUFA aa	1.17	0.31499	0.0423
PUFA aa	1.03	0.63053	0.0447
Tyrosine/Phenylalanine	0.97	0.63053	0.0470

In order to determine significance of each sum or ratio, observed *p*-values should be lower than the corrected alpha threshold. Legend: SFA: saturated fatty acids; MUFA: mono-unsaturated fatty acids; PUFA: poly-unsaturated fatty acids; UFA: unsaturated fatty acid, equal to the sum of MUFA and PUFA; Methionine-SO: sulfoxidized methionine; PC: phosphatidylcholine; aa: diacyl; ae: acyl-alkyl.

## Discussion

Our results show that the metabolome of embryonic rat cortical neurons is significantly impacted by siRNA-mediated decreased OPA1 expression as compared to controls. Taken as a whole, the OPA1 metabolomics signature is characterized by a decrease in aspartate, glutamate and threonine, and an increase in lysine and 42 glycerophospholipids.

The decrease in aspartate concentration was the most significant alteration identified by both univariate (*p* < 0.001) and multivariate (VIP > 1.9) analyses. This important metabolic feature associated to OPA1 silencing has also been found in our recent study of blood from OPA1 patients^25^ and in *Opa1*^*−/−*^ mouse embryonic fibroblasts^27^, thus in three samples from three different species. In contrast, the patient fibroblasts carrying heterozygous OPA1 pathogenic variants did not revealed a discriminant metabolomic signature^28^. Glutamate, the metabolism of which is tightly coupled to aspartate metabolism via the Krebs cycle and aminotransferases, was also reduced in siOPA1 neurons. This feature was also observed in patients and mouse embryonic fibroblasts. In the mouse model of OPA1 haploinsufficiency, aspartate concentration was invariant while glutamate concentration was respectively lower and higher in the optic nerve of symptomatic 11 month-old mice or asymptomatic 3 month-old mice^26^.

Two mechanisms could explain the alteration of aspartate homeostasis upon OPA1 deficiency. First, it has been reported that OPA1 directly interacts with the two mitochondrial aspartate/glutamate carriers (SLC25A12 and SLC25A13) involved in the exchange of aspartate and glutamate across the inner mitochondrial membrane^29^. Therefore, the decrease in OPA1 expression may alter the cytosolic-mitochondrial partitioning of glutamate and aspartate, and consequently their homeostasis. Second, aspartate deficiency has been shown to be a consequence of mitochondrial respiratory inhibition and impaired oxidative phosphorylation^30,31^. This would agree with the reduced mitochondrial membrane potential and the reduced expression of OXPHOS proteins that were previously reported in siOPA1 rat cortical neurons^16,21^ and more generally in OPA1 mutated conditions^10^. It should be noted that these two mechanisms are not mutually exclusive, because mitochondrial solute carriers use the inner membrane potential provided by OXPHOS as an energy supply for amino acids transport across this membrane.

Since aspartate is used as an amino-nitrogen donor during *de novo* glutamate synthesis in glial cells, the decrease in aspartate could lead to depletion in glutamate which in turn may impair synaptic transmission. Glutamate is indeed considered to be the main neurotransmitter of most excitatory synapses in the brain, through its interaction with the NMDA receptor (N-methyl-D-aspartate receptor). Interestingly, we have shown that synaptic plasticity is altered in siOPA1 neurons^21^ and others have shown that OPA1 expression influences NMDA receptor expression in the retina of a DOA mouse model^32^. Thus, the altered synaptogenesis plasticity related to OPA1 may be due to decreased NMDA receptor excitation as a consequence of aspartate/glutamate reduced concentrations. In turn, the lysine being a glutamate precursor through the saccharopine pathway in the mammalian central nervous system^33^, the impairment of glutamate biosynthesis may be responsible for the accumulation of lysine observed in the signature.

The increase in four sphingomyelins, four lysophosphatidylcholines and 34 phosphatidylcholines shows that OPA1 deficiency impacts considerably on phospholipidic composition of cortical neurons. These phospholipids represented a large proportion (42/105 = 39%) of the phospholipid species measured in the present targeted metabolomic analysis. Validating our results, such increased phosphatidylcholines was also confirmed in the same model using another mass spectrometer based lipidomic approach (data not shown). We previously reported that 12 phosphatidylcholines and one lysophosphatidylcholines also increased in the optic nerves of 11-month old *Opa1*^*−/−*^ female mice^26^. Also, in siOPA1 neurons identical to those used here, we previously showed that OPA1 silencing led to an alteration of the mitochondrial network that became highly fragmented^21^. The reconfiguration of phospholipid composition observed here may therefore reflect this fragmentation. In fact, the involvement of OPA1 in membrane fusion is mediated by a direct interaction with phospholipids in the inner mitochondrial membrane. Accordingly in yeast, Mgm1p, the OPA1 ortholog, promotes changes in phospholipid composition in liposomes to trigger membrane fusion^34^.

The phospholipid remodeling may also be linked to lipid droplets accumulating when mitochondrial fusion or OXPHOS activity is inhibited. Both the levels of triacylglycerols and the number and volume of lipid droplets increase upon OPA1 down-regulation in adipocytes and mouse embryonic fibroblasts^35,36^. Lipid droplets accumulation has also been found in the skeletal muscle of a mouse model with a conditional *Opa1*^+/−^ knock-out^17^. In addition, mitochondrial fusion is required in starved cells to redistribute lipid droplets-derived fatty acids across the mitochondrial network, to ensure optimal oxidation, and OPA1 dysfunction redirects fatty acids back to lipid droplets in both starved and non-starved cells^36^. In addition, it has been shown that a fraction of OPA1 protein ectopically localizes at the surface of lipid droplets in adipocytes, playing a direct role in the adrenergic control of lipolysis by anchoring the protein kinase A at the surface of lipid droplets^37^. Thus, the phospholipid signature we found here in siOPA1 neurons may simply result from lipid droplets that could accumulate, impacting fatty acids redistribution^36^ and/or lipolysis^37^.

The unsaturated to saturated diacyl- and acyl-alkyl phosphatidylcholine ratios also significantly decreased in siOPA1 compared to siLUC neurons, suggesting a general increase in the saturation of fatty acid chains of phosphatidylcholines and thus a decrease in the desaturases activities. This effect is likely related to oxidative stress that occurs in siOPA1 neurons^16^. In fact, both activity and expression of desaturases are regulated by the intracellular redox state^38^ and a low degree of fatty acid unsaturation may be a mechanism to protect cellular membranes from lipid peroxidation^39^.

Taken as a whole, our study shows that OPA1 deficiency in rat cortical neurons defines a highly specific metabolomic signature. It includes a general reconfiguration of glycerophospholipids, likely reflecting the alteration of mitochondrial fusion and the accumulation of lipid droplets. The second feature of the OPA1 signature in neurons was a sharp reduction in aspartate and glutamate concentrations, also found in other models of OPA1 dysfunction. This aspartate/glutamate signature is an attractive candidate to explain the neuronal specificity of the OPA1 clinical expression, through the perturbation of the NMDA receptor signaling.

## Supplementary information

Supplementary Table S1, Table S2, Figure S3

Suppl Table S4
