# Supplementary material for: The Metabolomic Signature of Opa1 Deficiency in Rat Primary Cortical Neurons Shows Aspartate/Glutamate Depletion and Phospholipids Remodeling
Source: Sci Rep. 2019 Apr 15;9:6107. doi: 10.1038/s41598-019-42554-7 (PMC6465244; doi:10.1038/s41598-019-42554-7)

THE METABOLOMIC SIGNATURE OF OPA1 DEFICIENCY IN RAT PRIMARY CORTICAL NEURONS SHOWS ASPARTATE/GLUTAMATE DEPLETION AND PHOSPHOLIPIDS REMODELING

Juan Manuel Chao de la Barca, Macarena S. Arrázola, Cinzia Bocca, Laetitia Arnauné-Pelloquin, Olga Iuliano, Guillaume Tcherkez, Guy Lenaers, Gilles Simard, Pascale Belenguer, Pascal Reynier

<table>
<tr><th colspan="3">Biochemical family</th><th>Number of metabolites</th><th>Method used for quantitation</th></tr>
<tr><td colspan="3">Carnitine (C0) and acyl-carnitines (AC)</td><td>40</td><td>FIA-MS/MS</td></tr>
<tr><td colspan="3">Amino acid (AA)</td><td>21</td><td>LC-MS/MS</td></tr>
<tr><td colspan="3">Biogenic amine (BA)</td><td>21</td><td>LC-MS/MS</td></tr>
<tr><td colspan="3">Hexose (H1)</td><td>1</td><td>FIA-MS/MS</td></tr>
<tr><td rowspan="4">Lipids</td><td colspan="2">Lysophosphatidylcholine (lyso PC)</td><td>14</td><td>FIA-MS/MS</td></tr>
<tr><td rowspan="2">Phosphatidylcholine (PC)</td><td>Diacyl-PC (PC aa)</td><td>38</td><td>FIA-MS/MS</td></tr>
<tr><td>Alkyl-acyl-PC (PC ae)</td><td>38</td><td>FIA-MS/MS</td></tr>
<tr><td colspan="2">Sphingomyelins (SM)</td><td>15</td><td>FIA-MS/MS</td></tr>
</table>

Supplementary Table 1. Biochemical families of metabolites quantified in Biocrates® Absolute IDQ p180 kit. FIA-MS/MS: Flow Injection Analysis-Tandem mass spectrometry. LC-MS/MS: Liquid Chromatography-Tandem mass spectrometry.

Ratio or sum	Metabolic significance
**Ratio of asymmetrically demethylated arginine to total unmodified arginine (ADMA/Arg)**	Inhibition of nitric oxide synthase (NOS)
**Ratio of total demethylated arginine to total unmodified arginine (Total DMA/Arg)**	Activity of protein arginine methyl transferase
**Ratio of citrulline to arginine (Cit/Arg)**	Activity of NOS
**Ratio of citrulline to ornithine (Cit/Orn)**	Activity of ornithine carbamoylphosphate transferase
**Ratio of ornithine to arginine (Orn/Arg)**	Activity of arginase
**Ratio of tyrosine to phenylalanine (Tyr/Phe)**	Activity of phenylalanine hydroxylase
**Ratio of sulfoxidized methionine to total unmodified methionine (Met-SO/Met)**	Measure of systemic oxidative stress
**Ratio of kynurenine to tryptophan (Kyn/Trp)**	Rate of tryptophan degradation to niacin (immunosuppression/tolerance)
**Ratio of serotonin to tryptophan (Serotonin/Trp)**	Rate of tryptophan degradation to serotonin
**Ratio of putrescine to ornithine (Putrescine/Orn)**	Activity of ornithine decarboxylase
**Ratio of spermidine to putrescine (Putrescine/ Spermidine)**	Activity of spermidine synthase
**Ratio of spermine to spermidine (Spermine/Spermidine)**	Activity of spermine synthase
**Ratio of acetylcarnitine to free carnitine (C2/C0)**	Measure of ß-oxidation of even numbered fatty acids (FA)
**Ratio of short chain acylcarnitines to free carnitine ((C2+C3)/C0)**	Measure of overall ß-oxidation activity
**Ratio of long chain acylcarnitines to free carnitine ((C16+C18)/C0)**	Activity of carnitine palmitoyltransferase I
**Ratio of dicaboxy-acylcarnitines to total acylcarnitines (Total AC-DC/Total AC)**	Indicator of ω-oxidation of FA.
**Sum of saturated FA in diacyl PC (SFA aa)**	Indicator of lipid composition on SFA aa
**Sum of saturated FA in acyl-alkyl PC (SFA ae)**	Indicator of lipid composition on SFA ae
**Sum of mono-unsaturated FA in diacyl PC (MUFA aa)**	Indicator of lipid composition on MUFA aa
**Sum of mono-unsaturated FA in acyl-alkyl PC (MUFA ae)**	Indicator of lipid composition on MUFA ae
**Sum of poly-unsaturated FA in diacyl PC (PUFA aa)**	Indicator of lipid composition on PUFA aa
**Sum of poly-unsaturated FA in acyl-alkyl (PUFA ae)**	Indicator of lipid composition on PUFA ae
**Unsaturated (MUFA+ PUFA) to saturated FA in diacyl PC (UFA/SFA aa)**	Measure of the activity of desaturases on diacyl PC
**Unsaturated (MUFA+ PUFA) to saturated FA in acyl-alkyl PC (UFA/SFA ae)**	Measure of the activity of desaturases on acyl-alkyl PC
**Ratio of lysoglycerophosphocholines to glycerophosphocholines (Total lysoPC/ Total PC)**	Indicator of phospholipase activity

Supplementary Table S2. Ratios or sums of metabolites selected for their potential metabolic significance in the setting of cultured cells.

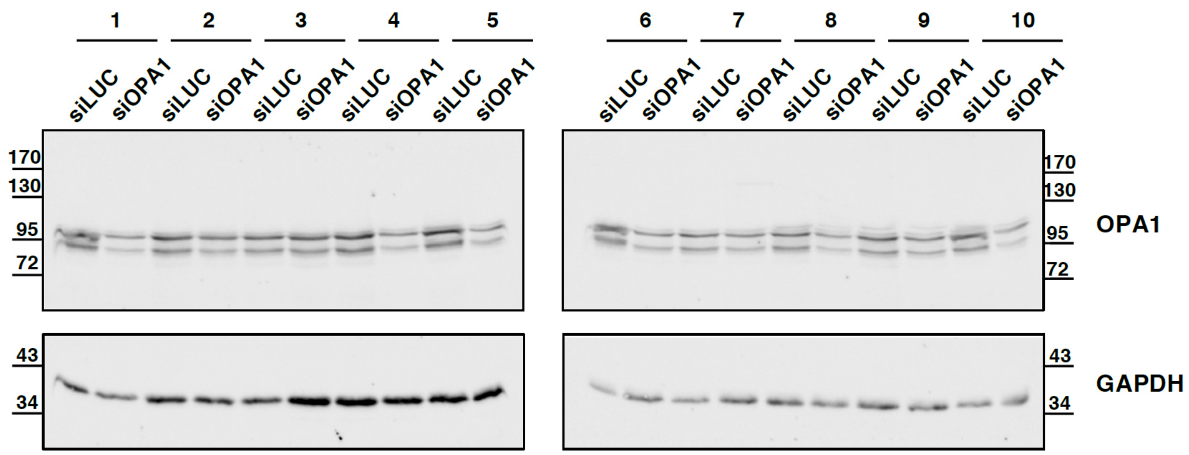

Supplementary Fig. S3: OPA1 (higher panel) and GAPDH (lower panel) cropped immunoblots showing at DIV9 the quantities of proteins in neurons transfected with siLUC or siOPA1 in 10 independent experiments. The two 9% polyacrylamid gels required for this analysis were processed in parallel and membranes were exposed with the same duration. The full length immunoblot is showed below.

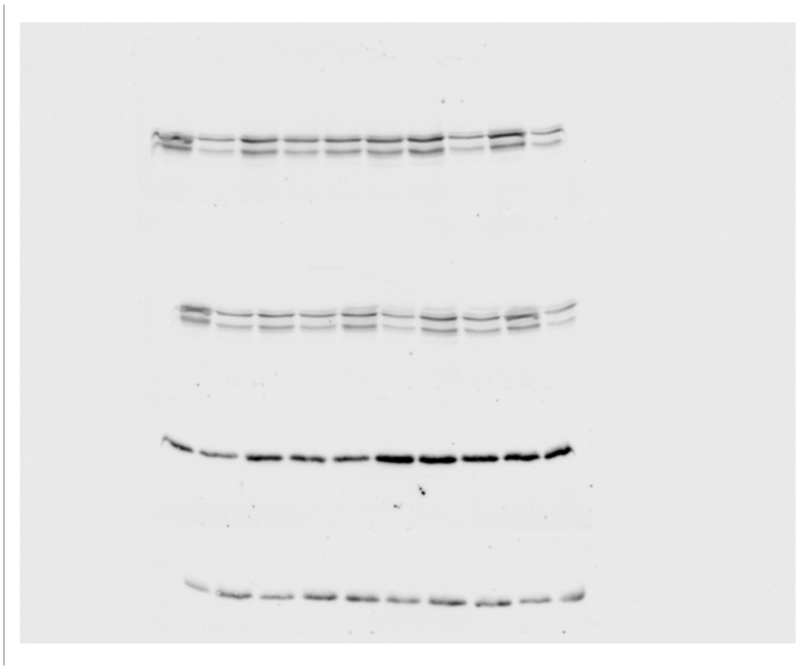

Supplement: Supplementary file 1 — Supplementary Table S1, Table S2, Figure S3 [file 41598_2019_42554_MOESM1_ESM.pdf]
